# Stimulation of Hyphal Ramification and Sporulation in *Funneliformis mosseae* by Root Extracts Is Host Phosphorous Status-Dependent

**DOI:** 10.3390/jof8020181

**Published:** 2022-02-11

**Authors:** Xueguang Sun, Jingwei Feng, Jing Shi

**Affiliations:** 1 Institute for Forest Resources & Environment of Guizhou, Guizhou University, Guiyang 550025, China; jingwei1392022@126.com; 2 Key Laboratory of Forest Cultivation in Plateau Moutain of Guizhou Province, Guizhou University, Guiyang 550025, China; 3 College of Forestry, Guizhou University, Guiyang 550025, China; 4 School of Sociology, Guizhou Minzu University, Guiyang 550025, China; sj008009@163.com

**Keywords:** arbuscular mycorrhizal fungi, *Funneliformis mosseae*, *Trifolium repens*, root exudates, root extracts, spore germination, secondary spore, metabolites

## Abstract

A simulation of the environment inhabited by arbuscular mycorrhizal (AM) fungi could provide clues as to how to cultivate these obligate biotrophs axenically. Host intraradical and rhizospheric environments, root extracts and exudates in particular, would be crucial for AM fungi to complete their life cycles. In this study, we analyzed and compared the effects of root exudates (RE) and root extracts (RET) of white clover (*Trifolium repens*) on the asymbiotic growth of the AM fungus *Funneliformis mosseae* in vitro, and furtherly analyzed the chemical components of different RET with the LC-MS/MS technique in order to establish an asymbiotic cultivation system for this important and hardly domesticated AM fungus. RET is superior to RE in stimulating spore germination, hyphal elongation and branching, and secondary spore formation (*p* < 0.05). RET-induced effects were dependent on phosphate supplement levels, and the RET obtained following the treatment with low levels of phosphorus significantly promoted hyphal growth and sporulation (*p* < 0.05). A few newly formed secondary spores showed limited colonization of white clover roots. The low phosphorus-induced effects could be ascribed to the metabolic adjustment (mainly lipids and organic acids) of white clover roots under low phosphate conditions. Our findings demonstrate that the low phosphate-induced RET boosts the asymbiotic growth of AM fungus, and thus offers an alternative way to fulfill the life cycle of AM fungi asymbiotically.

## 1. Introduction

Arbuscular mycorrhizal (AM) fungi are asexual obligate biotrophic symbionts that are dependent on carbon supplied by the roots of their host and are incapable of completing their life cycles asymbiotically [1]. Although a lot of research effort has being spent on obtaining pure cultures of AM fungi, a cultivation method that would enable most AM fungi to produce infection-competent spores in the absence of a host plant has not been reported to date.

A wide range of compounds (e.g., flavonoids, polyamines, cutin monomers, plant hormones, etc.) have been reported to promote AM fungal presymbiotic hyphal growth, branching and colonization efficiency [2,3,4,5,6]. Among these compounds, the branching factor, strigolactones (SLs), received the most concern, and they have been shown to greatly promote the hyphal ramification of AM fungi to initiate the establishment of AM symbiosis [7,8]. Although these compounds could promote the asymbiotic growth of AM fungi, there is no evidence that these stimulators could stimulate AM fungal sporulation and help fulfill their life cycles in vitro. Recently, the fatty acid auxotrophy of AM fungi was supported by studies showing that lipids synthesized by host plants are transferred to fungal symbionts and parasites, and that these fungi lack genes encoding cytosolic fatty acid synthases [9,10,11]. Kameoka et al. [12] and Sugiura et al. [13] assessed the effects of several fatty acids and lipids on the asymbiotic growth of AM fungi, trying to identify the fatty acids that promote AM fungal biomass production. They found that myristate (C14:0) [13] and palmitoleic acid (C16:1) [12] promoted the growth of several AM fungi, and particularly induced the formation of infection-competent secondary spores (SS) of *Rhizophagus irregularis*. However, no newly formed spores were observed in cultures of *Gigaspora margarita*. These results imply that the fatty acid preferences of AM fungi may be species-specific. Rather than a core compound determining the completion of AM fungi life cycles, it seems more likely that blends of compounds are needed for AM fungi to survive asymbiotically [6].

Simulating the natural environmental conditions that microbes dwell in is an approach that has been used to obtain pure cultures of microorganisms previously considered to be unculturable [14,15]. By constructing diffusion chambers that allowed the passage of substances from the natural environment (intertidal marine sediment) across a membrane, Kaeberlein et al. [14] and Nichols et al. [16] successfully grew bacteria from marine environments that were previously uncultivated. Similarly, simulating intraradical or rhizospheric environments inhabited by AM fungi could be a way of obtaining pure cultures of AM fungi. Among the many attempts, root exudates (RE) have been the most intensively investigated [17,18,19,20,21]. The presymbiotic growth of AM fungal spores is thought to be highly dependent on RE [22,23]. Host RE have been shown to increase hyphal length [19,20,24,25], stimulate hyphal branching [6,18,24,25,26,27] and facilitate the hyperpolarization of the fungal plasma membrane [28,29]. Compared with studies of RE, there have been few studies of the effects of root extracts (RET), especially of crude root aqueous extracts, on AM fungal spore germination or hyphal elongation. Although the stimulatory effects of some specific metabolites derived from RET (such as isoflavonoids [20,25]) on AM fungal growth and differentiation have been demonstrated, as a whole, with blends of metabolites, the effects of RET are still unknown. In AM–plant symbioses, AM fungi derive carbohydrates directly from roots. Given that plant roots generate plenty of primary and secondary metabolites, it seems reasonable to predict that RET may play more vital roles than RE in AM fungal development.

Mineral nutritional environments, especially nitrogen and phosphorus, play essential roles in regulating plant metabolism, which could further influence the relationships between plants and AM fungi [30,31]. AM formation can be stimulated under low phosphate conditions and hampered when supplemented with high levels of phosphate [32,33]. This could be at least partially explained by plant root metabolic changes under different exogenous phosphate supplies. It seems likely that the host plant accumulates metabolites that facilitate AM fungal growth under phosphate starvation and that inhibitory metabolites may accumulate in roots under high phosphate conditions. Thus, it would be intriguing to evaluate the effects of RET produced under different phosphate levels on AM fungal growth. AM responsiveness is known to be not only influenced by phosphate but also by other nutrients or by the combined effects of different minerals [34,35]. However, the effects of different nutrients and different nutrient levels on root metabolic regulation and, crucially, on the asymbiotic growth of AM fungi are largely unknown. Furthermore, whether different nutrients (particularly phosphate and nitrogen) have interactive effects on AM fungal growth is also unknown.

Based on the findings of previous studies, we hypothesize that: (i) the stimulation effects of RET on AM fungal development may be superior to those of RE; (ii) the effects of RET on AM fungal development are dependent on the host nutrition status. In this study, RE and root aqueous extracts of a wildly used and easily accessible AM fungal host, *Trifolium repens* [36,37,38,39], were prepared and their effects on the development in vitro of *Funneliformis mosseae*—an important, globally distributed, yet hardly domesticated AM fungus [40,41]—were evaluated and compared, aiming at offering a clue to obtain pure culture of this AM fungus.

## 2. Materials and Methods

### 2.1. AM Fungus and Plant Materials

*F. mosseae* (BGC NM01A), purchased from the Bank of Glomales in China, was cultured in pot cultures with maize (*Zea mays* L.), which acted as the host, and a silica sand substrate. Plants were grown in a greenhouse and Hoagland’s solution was applied weekly [42]. The substrate was harvested and air-dried after 6 months; spores of *F. mosseae* were collected using a wet sieving and decanting method [43]. Only mature spores (i.e., with a yellowish, full and glossy appearance), not aged spores, were selected and stored at 4 °C prior to the start of the experiments.

Seeds of white clover (*T. repens* L., cultivar Haifa, Evergreen Beijing International Grass Co., Ltd., Beijing, China) were surface sterilized with 98% sulfuric acid for 10 min and then washed three times with sterilized water. The sterilized seeds were then left to germinate on 0.8% water agar medium in Petri dishes at 25 °C. After the seeds had sprouted and the first leaf emerged, the plantlets were transplanted to a hydroponic culture system for RE or RET collection.

### 2.2. Experiment 1: Effects of RE versus RET on F. mosseae Growth

#### 2.2.1. RE and RET Preparation

A hydroponic culture method was used to cultivate white clover seedlings. Clean glass beads were placed in culture flasks (two seedlings per flask) and immersed in a liquid minimal (M) medium [44]. Seedlings were transplanted onto a cellulose membrane placed on the surface of the glass beads. The bottom of the flasks was covered with aluminum foil to reduce the adverse effects of light radiation on root growth. The plants were cultivated under a light/dark cycle with an illumination intensity of 150 µmol m^−2^ s^−1^ for 14 h a day at 25 °C followed by 10 h of darkness at 20 °C. The medium in each flask was replaced once a week using a disposable syringe.

After 4 weeks, the plantlets were carefully removed and their roots were washed twice with sterile distilled water before being transferred to new flasks containing 20 mL of sterile distilled water. The plantlets were cultured on a shaker (40 rpm) for three days (under the same cultivation conditions as those described above) before harvesting the RE by collecting the distilled-water solution in which the plantlets were growing. RE were adjusted to 5 or 50 mL/g fresh roots by adding sterile distilled water or concentrated using a rotary evaporator at a low temperature (below 35 °C) (1:5 and 1:50, respectively), and the pH was adjusted to 6.5 (around the middle of pH range of crude root exudates and extracts that we prepared; it is also the optimal pH for *F. mosseae* growth according to previous reports [45,46,47,48]). RE were then stored at 4 °C until ready for further use. After collecting RE, the roots were excised from stems, weighed in a sterile environment and then vigorously ground up in a precooled mortar and washed off with 5 mL of sterile distilled water. The mixture was centrifuged for 10 min at 10,000× *g* (4 °C) and the supernatant (RET) was recovered. RET were adjusted to 5 or 50 mL/g fresh roots by adding sterile distilled water (1:5 and 1:50, respectively) and the pH was adjusted to 6.5. RET were also stored at 4 °C. All the steps above were carried out under sterile conditions. 

#### 2.2.2. Effects of RE versus RET on *F. mosseae* Hyphal Growth 

The activity of exudates or extracts was assessed by performing a spore germination and hyphal branching assay. Spores were sterilized by immersion in a filter-sterilized distilled-water solution containing 5% (*v*/*v*) chloramine T, 50 mg/L chloramphenicol, 100 mg/L gentamicin sulfate, 200 mg/L streptomycin sulfate and 0.05% (*v*/*v*) Tween 20 for 20 min, and then rinsed in sterile distilled water three times. Petri dishes (70 mm) were half-filled with silica sand, which was then saturated with exudates, extracts or sterilized water (which acted as the control). Small discs (7 mm in diameter) of nitrous cellulose were cut using a single-hole paper punch. Nine discs were evenly distributed across the sandy surface of each Petri dish. A single *F. mosseae* spore was placed on each disc. Petri dishes were sealed with parafilm and incubated at 25 °C (optimal temperature for *F. mosseae* growth according to previous reports [45,46,47]) in the dark for 10 d. Data collected from 10 dishes were combined as one sample and each treatment had five replicates.

The hyphal length was assessed after cultivation for 10 d (hyphae were stained with 0.5% trypan blue for easy observation). Images of germinated spores magnified under a dissecting microscope (SZ61, Olympus, Tokyo, Japan) were obtained with a digital camera. The hyphal length in these images was measured using Image Pro-Plus 5.1 (Media Cybernetics Inc., Rockville, MD, USA). The number of hyphal branches and SS was also recorded directly by going through all the hyphae.

### 2.3. Experiment 2: Effects of Phosphorus and Nitrogen Status on Stimulatory Effects of RET on F. mosseae Growth

Four different M media treatments were used to assess the effects of phosphorus and nitrogen on the stimulatory effects of RET on *F. mosseae* growth. White clover seedlings were cultured in M medium as already described. M medium is low in phosphate (35 μM) and nitrogen (2 mM) and, hence, M medium was used for the low phosphate and low nitrogen treatment (LP + LN). For the high phosphate and low nitrogen treatment, the phosphate level was adjusted to 2 mM with KH_2_PO_4_ (HP + LN), whereas for the low phosphate and high nitrogen treatment, the nitrogen level was adjusted to 15 mM with KNO_3_ (LP + HN). Finally, for the high phosphate and high nitrogen treatment (HP + HN), the phosphate and nitrogen levels were adjusted to 2 mM and 15 mM with KH_2_PO_4_ and KNO_3_, respectively. 

After 4 weeks, the plant roots were washed twice with sterile distilled water, vigorously ground in a precooled mortar and then washed off with 5 mL of sterile distilled water. The mixture was centrifuged for 10 min at 10,000× *g* (4 °C). The supernatant was recovered and adjusted to 5 mL/g fresh roots by adding sterile distilled water. The effects of RET on *F. mosseae* spore germination and hyphal branching were assayed as described above.

To further assess the infectivity of secondary spores (SS), *F. mosseae* spores were incubated with RET on M medium (LP + LN) for two months. Every 15 days, the remaining RET solution was replaced with 10 mL of fresh RET using a sterilized syringe. Cultures were then divided into two parts: one part was used to perform an SS viability check; the other part was used to check the infection potential. The viability of SS was determined using the tetrazolium chloride vital stain INT (2-(p-iodophenyl)-3-(p-nitrophenyl)-5-phenyl-2H-tetrazolium chloride), following the procedure described by Walley and Germida [49]. Germinated spores were immersed in 2 mg/mL INT and incubated at 28 °C for 72 h before they were examined under a microscope (M205FA, Leica, Wetzlar, Germany). Spores with contents that stained red were considered viable. To check the infection potential of SS, SS were individually detached from mother spores to assess their germination viability following the procedures described above. An inoculation experiment was conducted using white clover seedlings (two weeks post germination) grown in sterilized sand using the sandwich method described by Giovannetti et al. [50]. Seedlings were cultivated for three months (using the same cultivation conditions mentioned above) before recovering the roots to evaluate the colonization rate of *F. mosseae* SS on roots according to the method described by Sun and Tang [51].

### 2.4. Experiment 3: Effects of Host Phosphorus Status on Root Metabolomes

Metabolomic analyses were performed on the roots of plants (from Experiment 2) subjected to LP + LN or HP + LN treatments. The roots were washed twice with sterilized distilled water. Roots of five flasks from the same treatment were pooled to form one sample, and for each treatment, three independent samples were collected. RET were obtained following the same procedure as described above.

RET was freeze-dried and extracted overnight at 4 °C with 1.0 mL of 70% aqueous methanol. Following centrifugation at 10,000× *g* for 10 min, extracts were absorbed (CNWBOND Carbon-GCB SPE Cartridge, 250 mg, 3 mL; ANPEL, Shanghai, China, www.anpel.com.cn/, accessed on 11th February 2022) and filtrated (SCAA-104, 0.22 μm pore size; ANPEL, Shanghai, China, http://www.anpel.com.cn/, accessed on 11 February 2022) before liquid chromatography mass spectrometry analysis.

Sample extracts were analyzed using a liquid chromatography–electrospray ionization–tandem mass spectrometry (LC-ESI-MS/MS) system (HPLC, Shim-pack UFLC SHIMADZU CBM30A system, www.shimadzu.com.cn/, accessed on 11 February 2022; MS, Applied Biosystems 6500 Q TRAP, www.sciex.com/products/mass-spectrometers/qtrap-systems/qtrap-6500plus-system, accessed on 11 February 2022). The following analytical conditions were used: HPLC, column, Waters ACQUITY UPLC HSS T3 C18 (1.8 µm, 2.14 mm × 100 mm); solvent system, water (0.04% acetic acid): acetonitrile (0.04% acetic acid); gradient program, 95:5 *v*/*v* at 0 min, 5:95 *v*/*v* at 11.0 min, 5:95 *v*/*v* at 12.0 min, 95:5 *v*/*v* at 12.1 min, 95:5 *v*/*v* at 15 min; flow rate, 0.40 mL/min; temperature, 40 °C; injection volume, 2 μL. The effluent was connected to an ESI-triple quadrupole-linear ion trap (Q TRAP)-MS.

Linear ion trap and triple quadrupole (QQQ) scans were acquired using a Q TRAP-MS, API6500 Q TRAP LC/MS/MS System, equipped with an ESI Turbo Ion-Spray interface, operating in a positive ion mode and controlled by Analyst 1.6.3 software (www.sciex.com/products/software/analyst-software, accessed on 11 February 2022). The ESI source operation parameters were as follows: ion source, turbo spray; source temperature, 500 °C; ion spray voltage, 5500 V; ion source gas I, gas II and curtain gas were set at 55, 60 and 25.0 psi, respectively; the collision gas was high. Instrument tuning and mass calibration were performed with 10 and 100 μmol/L polypropylene glycol solutions in QQQ and linear ion trap modes, respectively. QQQ scans were acquired as multiple reaction monitoring experiments with collision gas (nitrogen) set to 5 psi. The declustering potential and collision energy for individual multiple reaction monitoring transitions were set with further declustering potential and collision energy optimization. A specific set of multiple reaction monitoring transitions were monitored for each period according to the metabolites eluted within this period.

Differentially accumulated metabolites were screened by supervised orthogonal projection to latent structure discriminant analysis according to the default criteria of fold change being ≥2 or ≤0.5, and the variable importance in project (VIP) being ≥1 between LP and HP treatments. Each treatment had three biological replicates.

Identified metabolites were annotated using the KEGG Compound database (https://www.kegg.jp/kegg/compound/, accessed on 11 February 2022), annotated metabolites were then mapped to the KEGG Pathway database (https://www.kegg.jp/kegg/pathway.htm, accessed on 11 February 2022). Pathways with significantly regulated metabolites mapped to them were then analyzed using metabolite sets enrichment analysis; their significance was determined by calculating the *p*-values of a hypergeometric test.

### 2.5. Statistical Analysis

Data other than metabolic analysis data were analyzed using the statistical software SPSS 17.0.0 (Statistical Product and Service Solutions, SPSS Inc., Chicago, IL, USA). Prior to performing analyses, all data were checked for homogeneity of variance and normality of distribution. The data satisfied the assumption of homogeneity of variance. The effects of RE versus RET on *F. mosseae* growth were analyzed using one-way analysis of variance (ANOVA) and means were compared using Tukey’s HSD test (*p* < 0.05). The effects of phosphorus, nitrogen and their interaction on *F. mosseae* growth were analyzed using a two-way ANOVA; post-hoc comparisons were performed using Tukey’s HSD test (*p* < 0.05). Data are presented as means with their standard deviation. All experiments (metabolomes analysis conducted only once) were duplicated.

## 3. Results

### 3.1. Effects of RE versus RET on F. mosseae Spore Germination and Hyphal Ramification 

**Figure 1:**
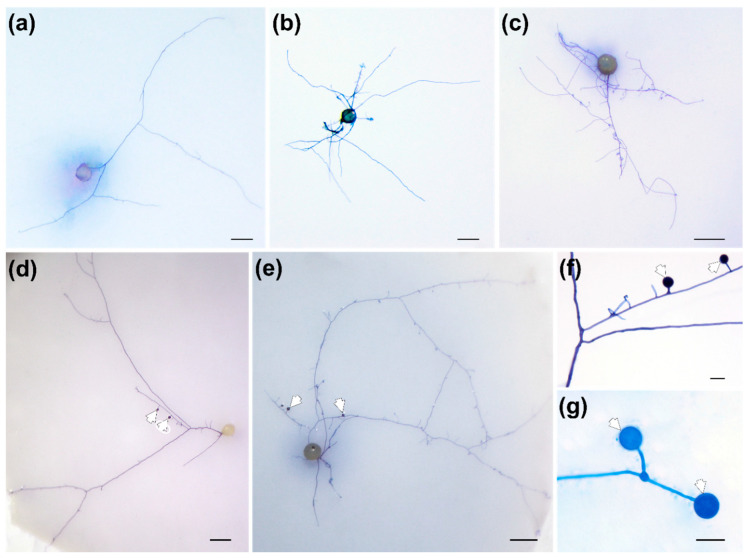
Effects of root exudates (RE) or root extracts (RET) on hyphal growth and differentiation of *Funneliformis mosseae*. Spores treated with sterilized water (control) (**a**), RE (1:50) (**b**), RE (1:5) (**c**), RET (1:50) (**d**) and RET (1:5) (**e**). Scale bars in (**a**–**e**) represent 200 μm. Solitary (**f**) and dichotomous (**g**) growth patterns of secondary spores (SS). Scale bars in (**f**,**g**) represent 20 μm. White arrowheads indicate SS.

RE and RET had differential effects on spore germination and hyphal elongation, ramification and differentiation of *F. mosseae* (Table 1 and Figure 1). Two weeks post cultivation, RE (1:5) significantly promoted spore germination and hyphal ramification (*p* < 0.05), whereas further dilution of RE (1:50) diminished these effects. Similar results were obtained with RET, with RET (1:5) performing better than more dilute solutions of RET (1:50) in stimulating spore germination, hyphal elongation and ramification. In particular, RET (1:5) greatly stimulated the formation of SS (*p* < 0.01). By contrast, for other treatments, fewer germinated mother spores were able to generate SS, and only between one and three SS per SS-forming mother spore were observed.

Germinated spores of *F. mosseae* had only one germ tube (Figure 1). RE (1:5) induced intensive branching, whereas RET (1:5) mainly promoted hyphal elongation (with lots of small side branches) (Figure 1a,c,e). SS were always produced solitarily, usually at the tips of thin branching secondary hyphae (Figure 1f), although sometimes a dichotomous growth pattern was observed (Figure 1g).

### 3.2. Effects of Phosphorus and Nitrogen Status on Stimulatory Effects of RET on F. mosseae Growth

RET of plants subjected to the low phosphate treatment significantly stimulated *F. mosseae* hyphal elongation and differentiation, whereas the nitrogen treatments had no such effects (Table 2). The spore germination rate, hyphal length, hyphal tips, ratio of spores forming SS and the number of SS observed when *F. mosseae* was subjected to RET obtained following LP + LN treatment were all higher than those observed for other RET treatments. There were no significant interactions between phosphorus and nitrogen treatments.

**Figure 2:**
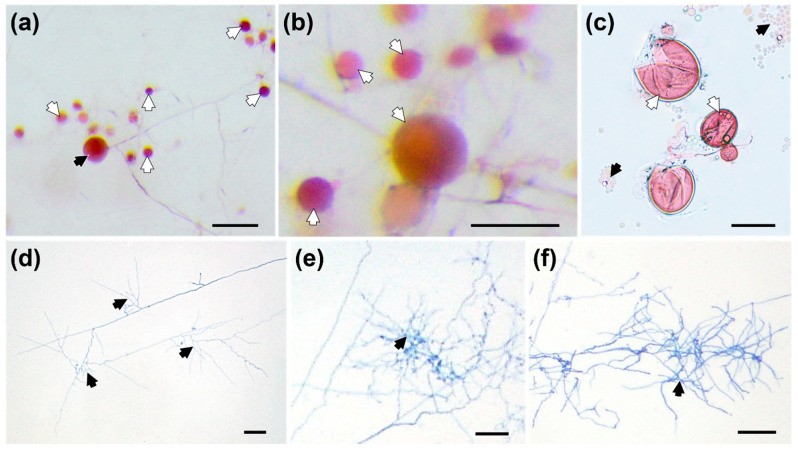
Secondary spores (indicated by white arrowheads) stained with the tetrazolium chloride vital stain INT to show their viability. The contents of viable spores are stained red (**a**–**c**). The black arrowhead in (**a**) indicates the germinating mother spore; the black arrowheads in (**c**) indicate lipids of crushed secondary spores. Germinated spores with branch absorbing structures (BAS, indicated by black arrowheads) (**d**–**f**). Scale bars in (**a**–**c**) represent 200 μm, 100 μm and 50 μm, respectively; scale bars in (**d**–**f**) represent 20 μm.

More viable SS formed under prolonged cultivation with RET of LP + LN-treated plants (Figure 2a–c). After two months, the average diameter of SS was 42.12 ± 17.89 μm (*n* = 50), which was significantly smaller than that of mature spores propagated with host plants (Figure S1). The largest SS were almost 90 μm in diameter (Figure 2b). The hyphae of germinated spores showed intensive branching and formed branch-absorbing structures (BAS) (Figure 2d–f).

**Figure 3:**
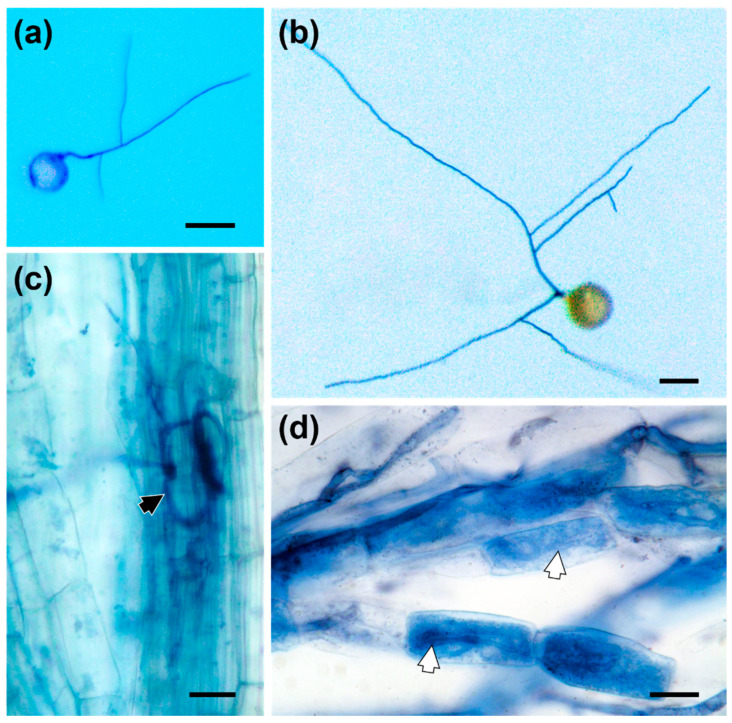
Germination and colonization potential of secondary spores (SS). (**a**) A germinating SS (10 d); (**b**) Germinated mother spore (10 d); (**c**) Intraradical hyphae (indicated by black arrowhead) formed by SS; (**d**) Arbuscular structures (indicated by white arrowheads) formed by the mother spore. Scale bars in (**a**,**b**) represent 100 μm; scale bars in (**c**,**d**) represent 50 μm.

Only a few SS germinated (<10%), and the hyphal growth of germinated SS was limited compared with that of the well-developed spores (Figure 3a,b). After coculture for three months, less than 20% of plants were colonized by SS, and few intraradical hyphae were observed (Figure 3c). However, more than 80% of plants inoculated with mature spores were successfully colonized and arbuscules were observed within root cortical cells (Figure 3d).

### 3.3. Effects of Phosphorus Status on Root Metabolomics

**Figure 4:**
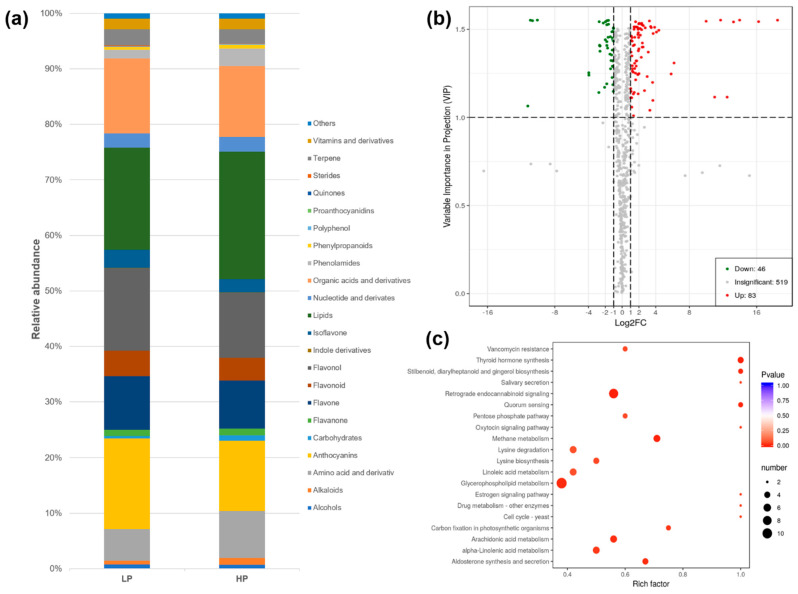
Metabolite categories of white clover roots grown under a high (HP) or low phosphorus (LP) treatment (**a**), differentially accumulated metabolites (LP versus HP) (**b**) and their KEGG annotations (**c**).

Metabolomics analysis of white clover RET identified 648 metabolites, which were further categorized into 22 classes (Table S1 and Figure 4a). Lipids, anthocyanins, flavonols and organic acids and derivatives were the most abundant metabolites, accounting for 60% relative abundance of all metabolites. 

The phosphorus fertilization regime greatly influenced the root metabolism of white clover, with the four largest classes of root metabolites all significantly changed under the HP treatment compared with under the LP treatment. Overall, 129 metabolites were differentially accumulated under LP vs. HP, with 46 downregulated and 83 upregulated (Figure 4b and Table S2). KEGG annotation revealed that these differentially accumulated metabolites are involved in several different biological processes, including glycerophospholipid metabolism and retrograde endocannabinoid signaling (Figure 4c).

**Figure 5:**
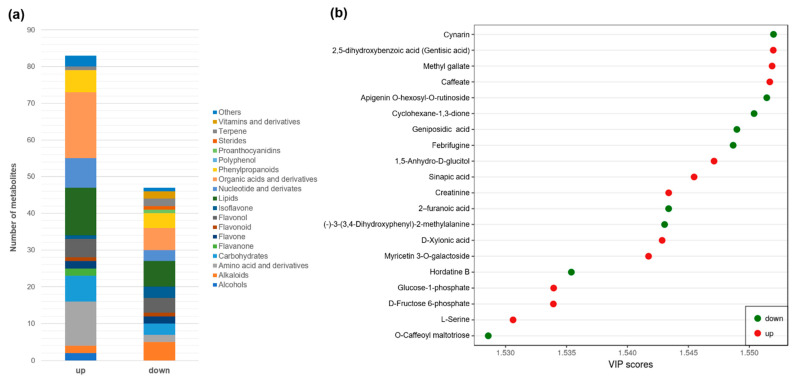
Classes of differentially accumulated metabolites of white clover roots grown under a low versus high phosphorus treatment (**a**) and the top 20 differentially expressed metabolites as shown by variable importance in projection (VIP) scores (**b**).

Collectively, upregulated metabolites were mainly organic acids and derivatives (18), lipids (13) and amino acids and derivatives (12) (Figure 5a). By contrast, the downregulated metabolites were mainly lipids (7), organic acids and derivatives (6) and alkaloids (5). Among the twenty metabolites with the highest VIP scores, nine were downregulated and eleven were upregulated (Figure 5b).

## 4. Discussion

### 4.1. Effects of RE versus RET on Hyphal Ramification and Differentiation of F. mosseae

The effects of RE on AM fungal growth could be controversial, with some studies showing positive effects, such as the formation of highly branched structures [18,26,27,52], while others only found a slight effect on hyphal elongation [6,19,47]. In this study, we did not observe significant hyphal elongation; however, more branches formed in the presence of RE compared with the control. Hyphal branching has been suggested as one of the events in host root recognition that precedes successful root colonization by AM fungi [27,53]. However, host RE cannot be the crucial factors that determine whether or not AM fungi are able to fulfill their life cycles asymbiotically because the stimulation effects of RE were limited and previous studies have shown no obvious difference in the stimulation of AM fungal development in vitro when using host or non-host RE [19,21,54,55,56,57].

Unlike RE, the effects of RET on the development of AM fungi in vitro are still unclear. Ishii et al. [58] found that the extracts of water-stressed bahia grass (*Paspalum notatum*) improved AM fungal growth, and later Horii et al. [59] isolated some compounds that promoted the growth of *G. margarita* and *Glomus caledonium* in water-stressed bahia grass roots. Flavonoids and isoflavonoids extracted from roots can stimulate hyphal growth of AM fungi even at trace amounts in vitro [20,25]. Here, we found that RET performed better than RE in stimulating AM fungal growth, such as the germination rate, hyphal length, hyphal branching and SS formation. An explanation for these findings might be the concentration of exudates/extracts used; however, a more likely explanation is that their metabolic components differ. For example, eupalitin, which stimulates AM fungi, has not been detected in bahia grass RE, but is present in the roots all year round [60]. Given that AM fungi colonize the host root and obtain organic substances from the root, it is clear that the root itself would play a more important role in the development of these fungi than its exudates.

Previous studies have reported that SS formation was observed while examining germinated spores of AM fungi and have suggested that plant secondary metabolites can influence their generation [61,62,63,64]. The newly formed spores are small, hyaline and hardly visible without staining, which may be the main reason why few studies have reported them. Hildebrandt et al. [63] and Abdellatif et al. [65] observed that SS of *R. irregularis* always formed at densely packed hyphal coils and were smaller than mother spores or spores formed in monoxenic culture with carrot root tissues. Similarly, in this study, SS that formed with root aqueous extracts were smaller than mother spores; however, they did not always form at highly packed hyphal coils, which may be due to the differentiation in AM fungal species.

### 4.2. Effects of Phosphorus and Nitrogen Status on Stimulation Effects of RET on F. mosseae Growth

RET of plants that received the low phosphate treatment significantly stimulated hyphal elongation and differentiation of *F. mosseae*, whereas the nitrogen treatments had no such effects. These results are unsurprising given that nitrogen has rarely been regarded as an important factor in determining root colonization by AM fungi [66]. We observed that the germinated spore of *F. mosseae* formed BAS when treated with RET of plants that received the low phosphate treatment, and a previous study designated BAS as a feature of the extraradical mycelium of symbiotic arbuscular mycorrhizal fungi [67]. Furthermore, the BAS may be involved in effective nutrient uptake by AM fungi [68]. The observation of BAS in this study indicates that the growth pattern of *F. mosseae* when supplemented with RET from the low phosphate treatment mimicked the growth pattern of AM fungi at the symbiotic stage. It is intriguing to find that a few germinated SS could limitedly (no arbuscules formed) colonize the roots of white clover. As a widely distributed AM fungal species, *F. mosseae* is hard to domesticate and cannot be continually propagated using the famous in vitro dual-culture system (i.e., cultivated using transformed root cultures), owing to a lack of sporulation [40,41]. Therefore, our findings at least offer an alternative route for propagating this fungus axenically in the future.

Metabolomic analysis revealed that phosphate levels greatly changed the accumulation of root metabolites, especially those of organic acids and derivatives, lipids and amino acids and derivatives. Similar results were obtained with other legumes such as alfalfa and soybean [69,70], indicating the homogeneously metabolic adjustments among plants (at least for the legume plants) when facing phosphate deficiency. These changed metabolites, especially lipids, could at least partially explain the different effects of phosphates on *F. mosseae* SS formation and growth, given that previous studies have repeatedly shown how vital lipids are for the survival of AM fungi [9,10,12,13].

In addition, there are some other metabolites that may promote the asymbiotic growth of *F. mosseae*. The accumulation of formononetin (an isoflavone) was significantly upregulated under the low phosphate treatment (Table S2), which supports the findings of Nair et al. [20]. This compound has been shown to stimulate spore germination, hyphal growth and root colonization [20,71,72]. Genistein, another low phosphate-induced isoflavone, has also been shown to play an important role in promoting AM symbiosis development in tobacco roots [73]. Similarly, studies have shown that AM fungi increase cynarin in the roots of host plants [74,75], and this compound was also significantly accumulated in host roots grown under low phosphorus condition in our study. These compounds may also play important roles in promoting AM fungal asymbiotic growth. 

There is a broad consensus that AM fungi are fatty acid auxotrophs and that they depend on lipids synthesized by the host plant [9,10,11]. However, we did not identify myristate or palmitoleic acid in RET of white clover, which have been shown to greatly promote asymbiotic growth and sporulation of *R. irregularis* [12,13]. Instead, some other lipids (including several fatty acids) were detected in the RET of white clover (Table S1), and these metabolites may also contribute to the promotion effects on *F. mosseae* growth. It seems likely that, just like those of some other fatty acids (18:1ω7 and 20:1Δ11) [76,77], the myristate- or palmitoleic-acid-induced effects may only be specific for some AM fungi and do not occur in all AM fungi species [6].

We did not identify SLs in the RET of white clover either, and this may be due to the quite low concentration of SLs in the plant root (usually on the order of pg/g fresh weight) and their hydrophobic feature (hardly soluble in aqueous root extracts) [78]. Actually, SLs cannot be the determining factors in stimulating *F. mosseae* sporulation in this study, and so far, no reports on the effects of these compounds on AM fungal sporulation could be found. Furthermore, SLs are even not necessary for AM formation [79,80].

## 5. Conclusions

Overall, our results demonstrate the promotion effects of RET on *F. mosseae* asymbiotic growth and infection-competent SS production, opening an alternative route for propagating AM fungi in future. However, the effects of RET may be AM fungal/host plant genus- and even species-specific. In addition, recent studies have shown that some rhizospheric bacteria and some AM fungal endobacteria may promote AM fungal development axenically (so called mycorrhiza helper bacteria, MHB) [65,81]. Some plant endophytes like dark septate endophytes (*Dreschlera* sp.) could also favor AM fungal growth [82]. Large-scale profiling of the effects of RET of different host plants and the combined application of RET and these beneficial microbes may assist with the development of AM fungal axenic propagation in the future.

## Figures and Tables

**Table 1:** Effects of root exudates or extracts on *Funneliformis mosseae* spore germination and hyphal differentiation.

Treatments	Spore Germination Rate (%)	Hyphal Length (mm)	Hyphal Tips	Formation of SS	
				Ratio of Germinated MS Forming SS (%)	Number of SS (per SS Forming Spore)
Control	48.88 ± 5.76 a	34.20 ± 6.83 a	23.20 ± 6.69 a	15.00 ± 6.08 a	1.80 ± 0.83 a
RE (1:5)	66.66 ± 6.21 b	50.80 ± 9.93 ab	43.80 ± 7.22 bc	20.55 ± 9.74 a	2.00 ± 1.00 a
RE (1:50)	53.88 ± 19.3 ab	36.40 ± 5.17 a	31.60 ± 8.68 ab	9.44 ± 7.24 a	1.60 ± 0.89 a
RET (1:5)	70.00 ± 8.42 b	113.80 ± 15.27 c	56.00 ± 12.18 c	90.00 ± 8.47 c	5.20 ± 0.83 b
RET (1:50)	55.55 ± 15.95 ab	61.00 ± 11.85 b	31.60 ± 11.84 ab	44.44 ± 8.56 b	2.40 ± 1.14 a
Significance (d.f. = 4)					
F	3.362	48.083	8.890	82.934	12.222
*p*	0.029 *	0.000 **	0.000 **	0.000 **	0.000 **

Notes: RE, root exudates; RET, root extracts; MS, mother spore; SS, secondary spore; values are presented as the mean ± the standard deviation, *n* = 5. Means followed by the same letter within a column do not differ significantly at *p* < 0.05 using Tukey’s HSD test. ** *p* < 0.01; * *p* < 0.05.

**Table 2:** Effects of root extracts on *Funneliformis mosseae* spore germination and hyphal growth.

Treatments		Spore Germination Rate (%)	Hyphal Length (mm)	Hyphal Tips	Formation of SS	
					Ratio of Germinated MS Forming SS (%)	Number of SS (per SS Forming Spore)
LP + LN		66.66 ± 13.02 b	131.20 ± 37.67 b	52.40 ± 8.56 b	81.67 ± 14.38 b	5.80 ± 0.84 b
LP + HN		55.55 ± 10.39 ab	103.20 ± 29.76 ab	53.00 ± 10.46 b	77.78 ± 14.96 b	5.00 ± 0.71 b
HP + LN		50.00 ± 2.77 a	78.60 ± 15.17 a	22.40 ± 5.94 a	45.56 ± 5.76 a	2.60 ± 1.14 a
HP + HN		52.77 ± 5.19 ab	63.40 ± 16.19 a	24.40 ± 5.81 a	52.22 ± 5.69 a	2.40 ± 1.14 a
Significance						
P level (d.f. = 1)	F	6.049	15.260	68.161	38.323	44.263
	P	0.026 *	0.001 **	0.000 **	0.000 **	0.000 **
N level (d.f. = 1)	F	1.111	3.336	0.134	0.078	1.316
	P	0.307 ns	0.087 ns	0.719 ns	0.784 ns	0.268 ns
P × N (d.f. = 1)	F	3.086	0.293	0.039	1.123	0.474
	P	0.098 ns	0.596 ns	0.846 ns	0.305 ns	0.501 ns

Notes: LP, root extracts of white clover grown in low phosphate medium; LN, root extracts of white clover grown in low nitrogen medium; HP, root extracts of white clover grown in high phosphate medium; HN, root extracts of white clover grown in high nitrogen medium; MS, mother spore; SS, secondary spore; values are presented as the mean ± the standard deviation, *n* = 5. Means followed by the same letter within a column do not differ significantly at *p* < 0.05 using Tukey’s HSD test. ** *p* < 0.01; * *p* < 0.05; ns, no significance.

## Data Availability

The original data presented in this study are included in the article/Supplementary Materials, further inquiries can be directed to the corresponding author.

## Supplementary Materials

The following are available online at https://www.mdpi.com/article/10.3390/jof8020181/s1, Figure S1: Diameter of mother spores (MS) and secondary spores (SS); Table S1: The metabolic profiles of white clover roots grown under different phosphorus fertilization levels; Table S2: Differentially accumulated metabolites of white clover roots grown under different phosphorus fertilization treatments.
