# Supplementary material for: Stimulation of Hyphal Ramification and Sporulation in Funneliformis mosseae by Root Extracts Is Host Phosphorous Status-Dependent
Source: J Fungi (Basel). 2022 Feb 11;8(2):181. doi: 10.3390/jof8020181 (PMC8876493; doi:10.3390/jof8020181)
Supplement: Supplementary file 1 [file jof-08-00181-s001.zip › jof-1524408-supplementary-Figure S1.pdf]

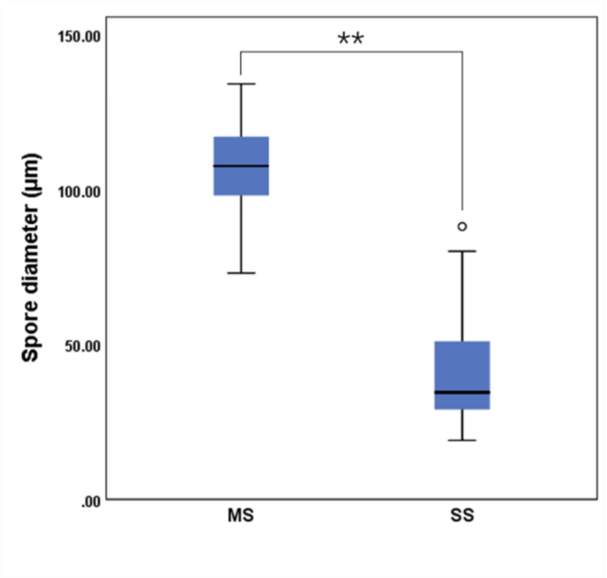

Figure S1. Diameter of mother spores (MS) and secondary spores (SS). ** indicates a significant difference in spore diameter between the two spore types at $P < 0.01$.
